# New Method of Treating Teeth after the Lining Membrane Has Become Exposed

**Published:** 1852-10

**Authors:** 


***New Method of Treating Teeth after the Lining Membrane has become Exposed.***—In August or September, 1851, Dr. S. P. Hullihen described to us a method which he had introduced of treating teeth after the lining membrane had become exposed. He stated that he had then practiced it for a considerable length of time, and with the most satisfactory results. We should have announced this new operation in the succeeding number of the Journal, but as the Doctor promised to furnish us with an article upon the subject, which should embody a description of his method of procedure, we did not wish to anticipate any thing which he might desire to say upon the subject. We heard nothing more from Dr. H. nor of the operation until after the last meeting of the American Society of Dental Surgeons, but soon after we were informed by a professional friend who was present on the occasion, that Dr. Cone had read a paper upon the subject, which embodied the history of the operation and a description of the manner of performing it, furnished by Dr. Hullihen. Subsequently a brief notice of the operation appeared in the Medical Examiner, under the name of E. B. Gardette, M. D., with a description of the manner of its performance.

The paper first alluded to has been published in the Dental News Letter, and the following is the description of the operation as given by Dr. Hullihen :

"The operation consists in making a hole through the gum, the outer edge of the alveolar process and root of the tooth into the nerve-cavity, and then in opening the blood-vessels of the nerve. The hole should be made of about the calibre of the nerve, at the point operated upon. If the drill employed be too large, there will be a difficulty in determining the exact moment when the nerve is reached. If too small, in obtaining the necessary discharge of blood. The drill should be spear-shaped, one cutting edge longer than the other, spring-tempered, and having a small neck. Spear-shaped, because the point is more easily located at the place desired. One cutting edge longer than the other, because such a shaped drill gives indication of its approach to the nerve-cavity, by catching in it, before it breaks through into the cavity. Spring-tempered, because less likely to break. Small necked, so as to permit the free escape of the cuttings made in the process of drilling. The operation may be commenced on either the incisors, cuspidatus, or bicuspids, by pushing the drill through the gum down to the alveolar process, about a line back from the edge of the process, and directly over the centre of the root of the tooth to be operated upon. Upon the molars, so that the hole will be freely opened upon the main body of the nerve. The drill is then driven forward by means of a very slack string and weak bow, until its near approach to the cavity is recognized by the catching sensation before mentioned. The drill and bow are now laid aside, and all the cuttings of the drill most certainly removed from the hole; then with a drill rotated with the fingers, the hole may be opened into the cavity. The friction of the drill upon the gum will prevent the bleeding from it. The entrance of the drill into the nerve-cavity usually opens the blood-vessels, which may at once be recognized by the color, (arterial blood,) and by the freedom of the discharge. By pressing a lock of cotton down into the carious cavity, an oscillation may be seen in the hole through the gum. By pressing the tooth into the alveolar cell, the bleeding may be much increased, either of which indications (so far as the making of the opening into the nerve-cavity is concerned) may be considered complete."

It appears from the communication from which the above is quoted, that Dr. Hullihen performed the operation as early as 1845, and having occasion fifteen months after to extract the tooth with a view to the application of an entire upper set of artificial teeth, he found the roots in a "perfectly healthy condition." As his patient had suffered no pain from the tooth subsequently to the operation, it was repeated on other teeth and with the most satisfactory results.

Dr. Gardette states that after having "fully tested" it "for more than a year," he has adopted it in his "own practice, where the condition of the tooth renders it proper."

With the above facts before us we were not a little surprised to see in the Boston Medical and Surgical Journal for October 20th, an article over the signature of Dr. S. P. Miller, in which this operation is described, and without a single allusion to Dr. Hullihen, with whom the operation originated. Dr. M., we believe, was present at the last meeting of the American Society of Dental Surgeons, where the paper already referred to was read, and even though the operation may have been performed by him without knowing that it had been practiced by H, for nearly five years, before he attempted it, he should, in justice to Dr. H., have stated the fact, and awarded to him the credit of being the first to originate, as well as the first to make it known to the profession.

